# Evasion of Innate Immune Responses by the Highly Virulent *Cryptococcus gattii* by Altering Capsule Glucuronoxylomannan Structure

**DOI:** 10.3389/fcimb.2015.00101

**Published:** 2016-01-06

**Authors:** Makoto Urai, Yukihiro Kaneko, Keigo Ueno, Yoichiro Okubo, Tomoko Aizawa, Hidesuke Fukazawa, Takashi Sugita, Hideaki Ohno, Kazutoshi Shibuya, Yuki Kinjo, Yoshitsugu Miyazaki

**Affiliations:** ^1^Department of Chemotherapy and Mycoses, National Institute of Infectious DiseasesTokyo, Japan; ^2^Department of Bacteriology, Osaka City University Graduate School of MedicineOsaka, Japan; ^3^Department of Surgical Pathology, Toho University School of MedicineTokyo, Japan; ^4^Department of Bioscience in Daily Life, College of Bioresource Sciences, Nihon UniversityKanagawa, Japan; ^5^Department of Microbiology, Meiji Pharmaceutical UniversityTokyo, Japan; ^6^Department of Infectious Diseases and Infection Control, Saitama Medical Center, Saitama Medical UniversitySaitama, Japan

**Keywords:** *Cryptococcus gattii*, *Cryptococcus neoformans*, capsule, glucuronoxylomannan, structure, *O*-acetylation, innate immune responses, dendritic cells

## Abstract

*Cryptococcus neoformans* causes life-threatening diseases mainly in immunosuppressed hosts such as AIDS patients; *C. gattii* causes disseminated infections even in healthy hosts. To identify the possible molecular mechanisms underlying this difference in virulence, we investigated the survival and histopathology of lung tissue in wild-type and CD4-depleted mice infected with *C. neoformans* H99 and *C. gattii* JP02 (the highly virulent strain isolated in Japan); we then compared dendritic cell (DC) cytokine release responses to different cell fractions from these two strains. JP02-infected mice exhibited shorter survival and fewer inflammatory cells in the lung than H99-infected control mice. Depletion of CD4-related cellular immunity reduced survival of H99-infected mice but had no effect on the survival or inflammatory cell infiltration in JP02-infected mice, suggesting that JP02 evades immune detection. To identify the molecule(s) conferring this difference, we measured cytokine production from murine DCs co-cultured with H99 and JP02 *in vitro*. The levels of inflammatory cytokines from DCs treated with intact JP02 cells, the extracted capsule, secreted extracellular polysaccharides, and purified glucuronoxylomannan (GXM) were markedly lower than those induced by intact H99 cells and corresponding H99 fractions. Structural analysis of GXM indicated that JP02 altered one of two *O*-acetyl groups detected in the H99 GXM. Deacetylated GXM lost the ability to induce inflammatory cytokine release from DCs, implicating these *O*-acetyl groups in immune recognition. We conclude that the highly virulent *C. gattii* processes a structural alteration in GXM that allows this pathogen to evade the immune response and therefore elimination.

## Introduction

Life-threatening diseases caused by *Cryptococcus neoformans* in immunosuppressed hosts such as AIDS patients occur at a global rate of approximately 1 million cases per year (Park et al., 2009). In contrast, infection by its sibling species *C. gattii* is much less common in AIDS patients but is thought to be more virulent than *C. neoformans* and causes disseminated infections even in healthy hosts (Galanis et al., 2009). Since 1999, more than 300 *C. gattii* infections have been reported in British Columbia and these cases were generally more severe than cryptococcosis due to *C. neoformans*, including a case of cerebral cryptococcoma (Galanis et al., 2010; BC Centre for Disease Control, 2015). So far, the difference in virulence between conventional cryptococcal pathogens and hypervirulent strains is under-investigated.

The difference in virulence between *C. neoformans* H99 and *C. gattii* R265, (the *C. gattii* strain was clinically isolated during the Canadian outbreak, Kidd et al., 2004) was characterized in previous studies. One study in CD4C/HIV^MutA^ transgenic mice showed that HIV-1 transgene expression consistently augments susceptibility to H99, but not to R265 infection and reduces the pulmonary inflammatory cell response by both depletion of immune cells and diminished production of chemokines (Leongson et al., 2013). Cheng et al. found that neutrophil infiltration and inflammatory cytokine production were lower in the lungs of wild-type mice infected with R265 compared with mice infected with H99 (Cheng et al., 2009). Accumulation of immune cells in the murine lung was lower in response to R265 infection than H99 infection (Okubo et al., 2013).

In the infectious state, both H99 and R265 form a thick capsule (Leongson et al., 2013) thought to be important for resistance against host innate immunity, such as evasion of phagocytosis (Buchanan and Murphy, 1998; Zaragoza et al., 2009). Acapsular strains of *C. neoformans* are less virulent than encapsulated ones because acapsular strains are directly exposed to the cell surfaces of immunocytes and thus strongly induce host immune responses (Buchanan and Murphy, 1998; Zaragoza et al., 2009). However, the factors responsible for the differences in immune response between highly encapsulated *C. neoformans* and *C. gattii* are still unknown.

Cryptococcal capsule is composed primarily of glucuronoxylomannan (GXM), which comprises more than 90% of the capsule's polysaccharide mass (Zaragoza et al., 2009). It is also secreted abundantly into the ambient environment (Cherniak and Sundstrom, 1994). The roles of GXM in cryptococcal virulence have been reported in *C. neoformans*. The GXM is important to abolish host immunity by inhibiting binding of antibodies and complement C3 (Young and Kozel, 1993; Cherniak and Sundstrom, 1994). On the other hand, it has been also shown that GXM can induce expression of inflammatory cytokines, such as TNF-α and IL-6, from human immune cells (Delfino et al., 1996, 1997; Retini et al., 1996; Walenkamp et al., 1999). However, the differences in GXM structure between *C. neoformans* and highly virulent *C. gattii* are still unclear.

The *C. gattii* strain JP02 was isolated together with JP01, the first genotype VGIIa strain isolated in Japan, from the same patient in 2007 (Okamoto et al., 2010). The strain JP02 belongs to the genotype VGIIc, and its virulence against mice is higher than R265 (our unpublished data). In the present study, we first compared virulence and induction of protective inflammation between *C. gattii* JP02 and *C. neoformans* H99 in both wild type and mice depleted of CD4-positive cells (a model of immune compromise) using an anti-CD4 antibody. Furthermore, we identified one capsule constituent, GXM, likely involved in determining the difference in murine dendritic cell immune response between JP02 and H99 cells by comparing *in vitro* cytokine release in response to intact cells and various subcellular fractions.

## Materials and methods

### Ethics

All experiments on mice were conducted according to the protocols approved by the Animal Care and Use Committee of the National Institute of Infectious Diseases, Japan (approval number 114029, 213054, and 215046).

### Strains

*C. neoformans* H99 and *C. gattii* JP02 were routinely cultured in yeast extract-peptone-dextrose medium (YPD, BD) at 30°C. To obtain the capsular polymeric substances (CPS) and extracellular polysaccharides (EPS), cells were grown in modified yeast nitrogen broth (mYNB) [YNB without amino acids (BD) supplemented with complete supplement mixture (FORMEDIUM) and 0.5% (wt/vol) of D-glucose].

### Mice

C57BL/6 female mice were obtained from Japan SLC (Shizuoka, Japan) and maintained under specific pathogen-free conditions at the National Institute of Infectious Diseases.

### Dendritic cells

The JAWSII cell line (ATCC® CRL-11904^TM^) derived from the bone marrow of the p53 KO C57BL/6 mouse was obtained from the American Type Culture Collection. Isolation of primary dendritic cells derived from the bone marrow (BMDCs) of female C57BL/6J mice was described previously (Ueno et al., 2015).

### Infection study

Eight-week-old mice were injected intraperitoneally with 300 μg monoclonal anti-CD4 antibody (purified anti-mouse CD4 antibody clone GK1.5, Biolegend, MA) or control antibody (purified Rat IgG2b, κ Isotype Ctrl, Biolegend) on the day prior to pathogen inoculation and with 100 μg on days 6, 13, and 20. Strains H99 and JP02 were cultured in YPD medium overnight, recovered by centrifugation, and resuspended in PBS. Mice were anesthetized with isoflurane, and 50 μl of a fungal suspension containing 5.0 × 10^3^ or 5.2 × 10^3^ cells was injected intratracheally using a 24-gauge indwelling needle (TOP Corporation, Japan). For survival analysis, mice were monitored daily for 34 days after infection. For histopathological examination and to determine fungal burden, mice were euthanized by carbon dioxide inhalation 2 weeks after infection and the lungs were harvested. Lungs were homogenized using a nylon mesh (70 μm; BD Falcon, NJ.) in 2 ml of PBS. The homogenates were diluted and spread onto YPD plates. The plates were incubated at 30°C and cryptococcal colonies were counted at 24 h post incubation. Histological analyses were performed as described previously (Okubo et al., 2013).

To confirm whether CD4+ T cells were depleted by anti-CD4 antibody treatment, flow cytometry analysis was performed. The blood was sampled from orbital sinus of mice on 6 days before and 2, 6, and 13 days after the first administration of monoclonal anti-CD4 antibody or control antibody. Red blood cells were lysed with lysis buffer [9 volumes of 0.83% (wt/vol) NH_4_Cl and 1 volume of 200 mM Tris-HCl, pH 7.6]. Residual cells were then stained for flow cytometry analysis. All antibodies and buffer used for cell staining were from Biolegend. Fc receptors were blocked with an anti-CD16/32 antibody (clone 93) and then, cells were stained with anti-CD4 (clone RM4-4) and anti-TCRβ (clone H57-597) antibodies. Data were acquired with a BD FACSCalibur™ flow cytometer (BD Bioscience) and analyzed by using FlowJo software (TreeStar Inc.).

### Isolation of CPS and EPS fractions, and purification of glucuronoxylomannan (GXM) from the EPS fraction

The H99 and JP02 strains were grown in mYNB at 30°C with shaking for 5 days. Cryptococcal cells were killed by heating at 105°C for 30 min and EPS was extracted from the culture medium supernatant as described previously (Urai et al., 2006), except using the Sevag method instead of phenol–chloroform treatment (Staub, 1965). The CPS fraction was extracted after EPS extraction as described previously (Bryan et al., 2005), with some modifications. Briefly, cells were delipidated by the Forch method (Folch et al., 1957), and capsule components extracted from the remaining cellular material using dimethyl sulfoxide. After centrifugation, supernatants were collected and dialyzed against MilliQ water.

GXM was purified from the EPS fraction by DEAE-Toyopearl 650 M column chromatography as described previously (Urai et al., 2007). Fractions containing saccharides were monitored by the phenol–H_2_SO_4_ method (DuBois et al., 1956) and dialyzed against MilliQ water.

EPS extraction from homogenate of murine lung infected with cryptococci followed the same procedure as described above with the exception of dialysis.

### Measurement of cytokine release from dendritic cells

JAWSII cells (2 × 10^5^ cells/mL) were incubated with intact cryptococcal cells, CPS, EPS, or GXM in minimum essential medium Eagle α (Sigma) with complete supplement mixture [10% (vol/vol) heat-inactivated fetal bovine serum (FBS, EQUITECH BIO), 1% (vol/vol) penicillin-streptomycin-L-glutamine solution (Wako; 10,000 units/ml penicillin G, 10 mg/ml streptomycin, 29.2 mg/ml L-glutamine, and 8.5 mg/ml NaCl), 1% (vol/vol) 200 mM L-glutamine solution (Wako), 1% (vol/vol) 100 mM sodium pyruvate solution (Gibco BRL), and 5 ng/ml mouse granulocyte-macrophage colony-stimulating factor (mGM-CSF, PeproTech Inc.) at 37°C under 5% CO_2_ for 24 h. Culture medium was harvested and centrifuged, and the supernatants were subjected to cytokine production assays as described below. The procedures for analysis of cytokine release from DCs derived from bone marrow (BMDCs) (1 × 10^6^ cells/mL) were similar except that cells were cultured and treated in complete RPMI 1640 medium (Sigma) supplemented with 10% (vol/vol) FBS, 1% (vol/vol) streptomycin-penicillin solution (Sigma; 10,000 units penicillin and 10 mg/ml streptomycin), 44 μM 2-mercaptoethanol, and 10 ng/ml mGM-CSF (PeproTech Inc.).

Live cryptococcal cells were collected by centrifugation from overnight culture in YPD medium and resuspended in PBS. Heat-killed cryptococcal cells were prepared by heating the cell suspension in PBS to 65°C for 1 h.

Release rates of IL-6, IL-12p40, and TNF-α were measured using Becton Dickinson Laboratory murine IL-6, IL-12p40, and TNF-α enzyme-linked immunosorbent assay (ELISA) kits according to the manufacturer's instructions. Culture medium of non-treated dendritic cells was used as the control of cytokine production.

### Structural analysis of GXM

Monosaccharide analysis was performed as described previously (Urai et al., 2006). Briefly, GXM was hydrolyzed by 4 M TFA at 100°C for 3 h, and the monosaccharides obtained were labeled with aminobenzoic acid ethyl ester (ABEE) and analyzed by high performance liquid chromatography (HPLC) (1500 HPLC system, Waters, Milford, MA). All nuclear magnetic resonance (NMR) spectra were recorded at 500 MHz (^1^H) with an ECA 500 instrument (JEOL Ltd. Tokyo, Japan). Chemical shifts are given in δ units with acetone (δ^1^H 2.23) used as an external reference for samples measured in D_2_O solutions. O-deacetylation and sonication of GXM were performed as described previously (Urai et al., 2006).

### Statistical analysis

SPSS software, version 20.0 (IBM Japan, Tokyo, Japan), was used for all statistical analyses. Normality of distribution and equality of variance were assessed using the Shapiro-Wilk and Levene tests, respectively. *P* values < 0.01 were considered significant.

## Results

### JP02 shows higher virulence than H99 in a murine pulmonary infection model

**Figure 1:**
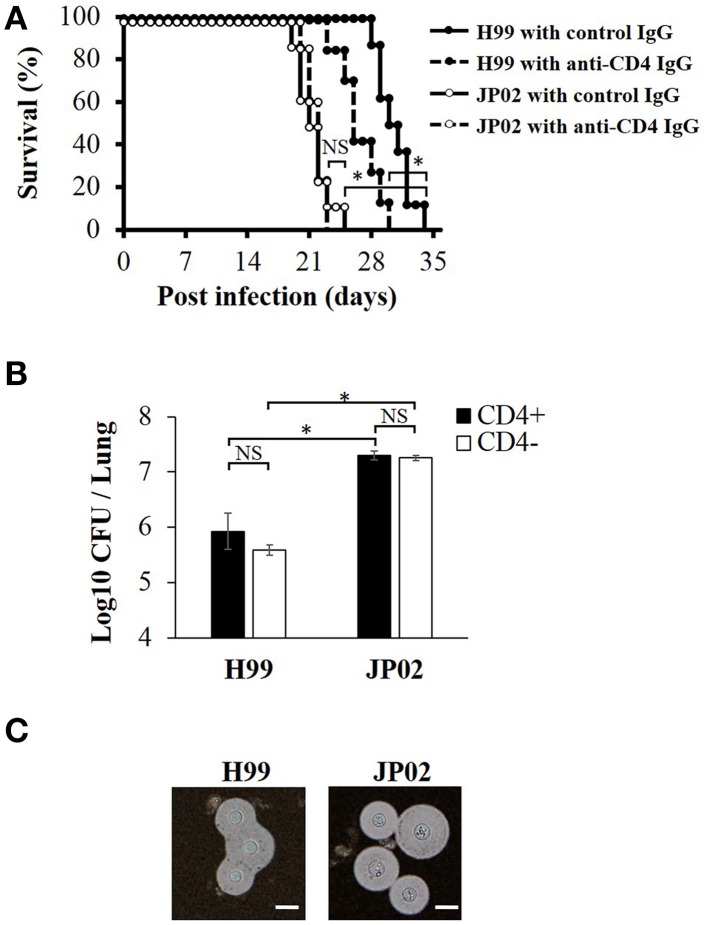
**Greater virulence and mortality of ***Cryptococcus gattii*** JP02 infection compared to ***Cryptococcus neoformans*** H99 infection and independence from CD4 cell-mediated immunity**. **(A)** Shorter survival of mice infected with JP02 compared with H99-infected mice (*n* = 8). Some mice were depleted of CD4-positive cells by an anti-CD4 injection before infection. The survival from H99 infection was shorter in CD4-depleted mice compared with that in CD4-positive control mice, while CD4 depletion had no effect on the survival from JP02 infection. **(B)** Fungal burden was substantially higher in the lungs of JP02-infected mice compared with the lungs of H99-infected mice. Results are representative of two individual experiments with *n* = 5 for each experiment (means ± standard deviations, SD). **(C)** Capsule formation as evaluated by India ink staining of infected lung homogenates showing thick capsule formation by both species. Bar, 10 μm. Survival curves were compared by log-rank test and the means of fungal burden were compared by the unpaired *t*-test. ^*^P < 0.01; NS, not significant.

To identify the possible mechanisms conferring the higher virulence of *C. gattii* JP02 compared to *C. neoformans* H99, we first measured survival rates of C57BL/6J mice following intratracheal pathogen injection. Control mice infected with JP02 exhibited significantly shorter median survival than those infected with H99 (21 vs. 30 days) (Figure 1A). The number of yeast cells recovered from the lung tissue of JP02-infected mice was 10 times higher than that recovered from H99-infected mice (Figure 1B). Both H99 and JP02 formed thick capsules as revealed by microscopic observation of India ink-stained lung homogenate (Figure 1C), so capsule formation does not appear to account for the difference in virulence between strains.

### Survival from H99 infection was reduced in CD4-depleted mice, while CD4(+) cell-mediated immunity had no effect on JP02 infection

To model infection under cellular immune deficiency, such as that associated with HIV infection, CD4-positive cells were depleted by injection of anti-CD4 antibody. The CD4+ cell depletion was confirmed by the flow cytometry analysis (Figure S1). These CD4-depleted mice exhibited significantly shorter survival following H99 infection than control H99-infected mice, while there was no significant difference in survival between CD4-depleted and non-depleted mice following JP02 infection (Figure 1A). These results suggest that H99 causes more severe infection in immunecompromised hosts while JP02 can cause severe infection even in immunocompetent hosts. Furthermore, suppression of CD4T cell-mediated immunity had no effect on survival or histopathologic signs of lung inflammation (below), suggesting that JP02 may not be recognized by the cell-mediated immune system.

### Inoculation with JP02 induced a weaker protective inflammatory response in the murine lung than H99

**Figure 2:**
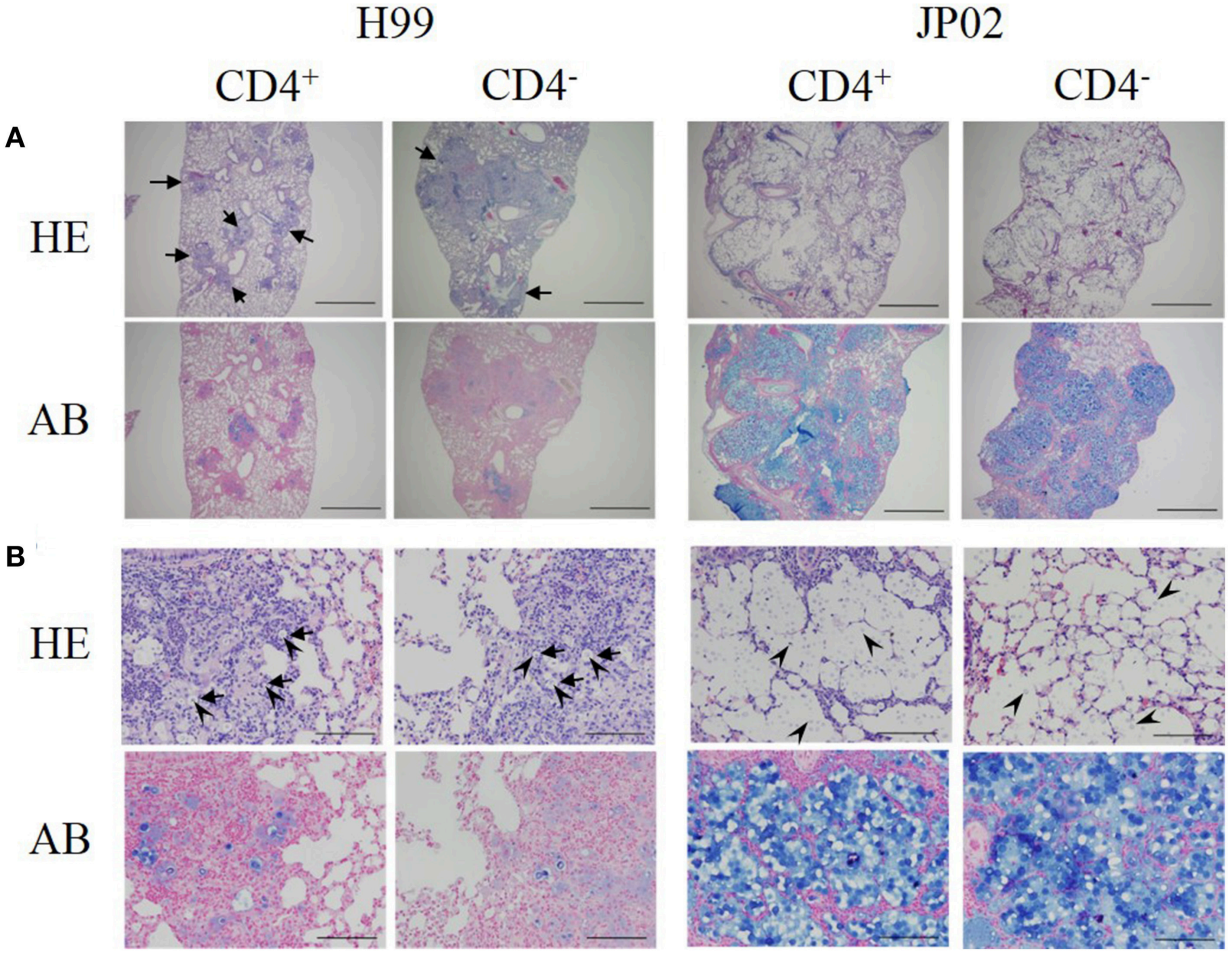
**Histopathological differences between H99- and JP02-infected lungs suggest less protective inflammation induced by JP02**. Lung paraffin sections were prepared from mice on day 14 after intratracheal infection, stained with hematoxylin-eosin (HE) or alcian blue (AB), and examined at magnifications of **(A)** × 40 (bar, 1 mm) and **(B)** × 400 (bar, 100 μm) under a light microscope. **(A)** Lung sections of control and CD4-depleted mice infected with H99 showed well-demarcated nodular lesions (arrows), but these were enlarged in CD4-depleted mice. In contrast, neither the lung sections of control nor CD4-depleted mice infected with JP02 exhibited nodular lesions, but both showed eccentric pulmonary enlargement and alveolar airspace variation. **(B)** Lung sections of control and CD4-depleted mice infected with H99 showed numerous MGCs (arrows). Yeast cells (arrow heads) were observed only in the nodular lesions, and these surfaces were well-stained by alcian blue. In contrast, the lung sections of control and CD4-depleted JP02-infected mice exhibited no inflammatory cell infiltration. Numerous yeast cells were observed in the alveoli, and its airspaces were filled with large amounts of an acidic substance stained by alcian blue.

Low-power fields of lung sections from control mice infected with H99 showed multiple well-demarcated nodular lesions but no alveolar airspace variation (Figure 2A). The lung sections of CD4-depleted mice infected with H99 also showed well-demarcated nodular lesions, but these were enlarged compared to control H99-infected mice. In contrast to H99-infected mice, CD4 depletion did not affect the histopathological features of murine lung infected with JP02. Neither lung sections from control JP02-infected mice nor CD4-depleted JP02-infected mice exhibited nodular lesions, but both showed eccentric pulmonary enlargement and alveolar airspace variation.

High-power fields of pulmonary sections from control mice infected with H99 showed numerous multinucleated giant cells (MGCs) with well-developed nuclei (Figure 2B). Yeast cells were observed only in the nodular lesions, and the surfaces of yeast cells were well-stained by alcian blue. These results suggest that H99 cells are recognized by host immune cells despite encapsulation. Sections of CD4-depleted murine lung infected with H99 showed MGCs with somewhat foamy cytoplasm and a smaller number of nuclei. However, yeast cells were still observed only in the nodular lesions. In contrast, pulmonary sections from both control and CD4-depleted JP02-infected mice exhibited no inflammatory cell infiltration. No MGCs were observed in sections from either CD4-depleted or non-depleted JP02-infected lungs. Numerous yeast cells with surfaces well-stained by alcian blue were diffusely spread throughout the alveoli. Furthermore, alveolar airspaces of JP02-infected murine lung were filled with large amounts of an acidic substance, probably GXM secreted from JP02 cells as revealed by alcian blue staining (Figure 2B). Thus, the JP02 strain did not appear to be recognized by lung immunocytes. Since capsule formation is thought to be critical for resistance against host innate immunity, we speculated that some capsule component present and/or secreted by JP02 but not H99 cells confers resistance against recognition by immune cells.

To assess this possibility, we first estimated the amount of secreted EPS from H99 and JP02 cells in murine lung using the phenol–H_2_SO_4_ method against polysaccharides extracted from lung homogenate. Consistent with the results of alcian blue staining, a greater amount of polysaccharide was detected in the homogenate of JP02-infected murine lung compared to H99-infected lung (Table S1). These results indicate that JP02 cells secrete a larger amount of GXM in murine lung, which may interfere with recognition by host immune cells.

### H99 induces greater interleukin-6 release from murine dendritic cells than JP02

**Figure 3:**
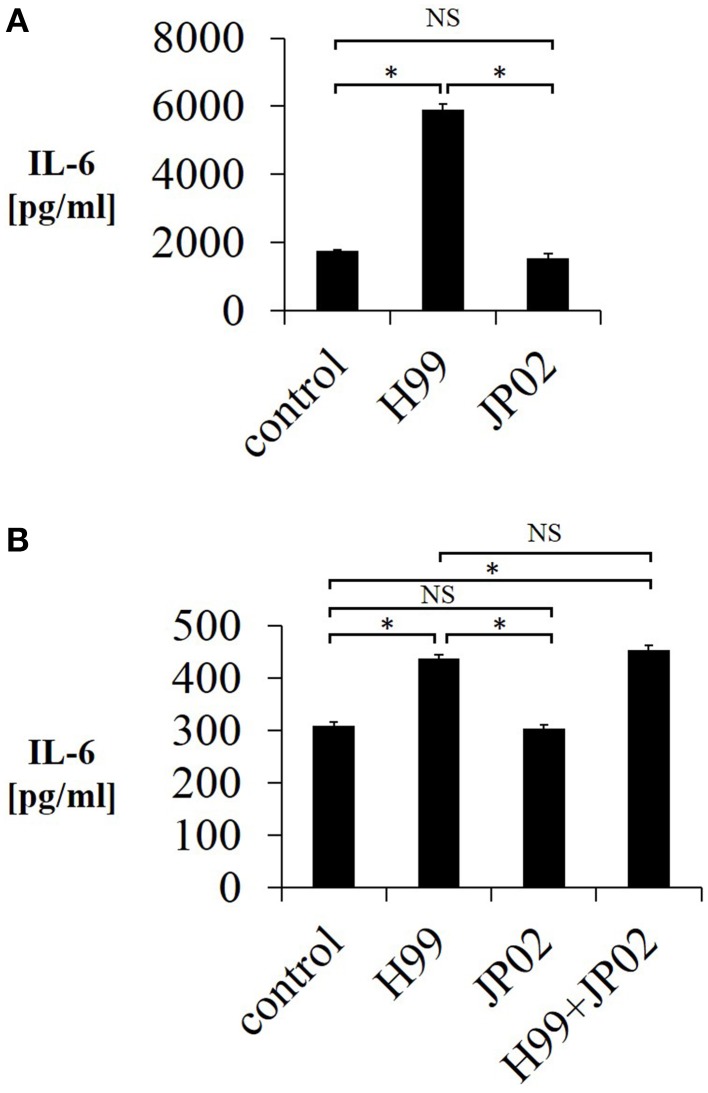
**Lower IL-6 release from JAWSII dendritic cells in response to live or heat-killed JP02 cells compared with live or heat-killed H99 cells**. JAWSII cells (2 × 10^5^ cells/mL) were incubated with live (MOI = 1) or heat-killed (MOI = 5) cryptococcal cells for 24 h, and supernatant was obtained for ELISA. **(A)** Incubation with live cells. **(B)** Incubation with heat-killed cells. H99 + JP02 means co-incubation with heat-killed H99 and heat-killed JP02 cells (MOI = 5, each). The experiments were performed in triplicate (means ± SD). Treatment group means were compared by the unpaired *t*-test, except for vs. H99 in **(B)**, which were compared by the Mann–Whitney U-test. ^*^P < 0.01; NS, not significant.

One possible reason for the shorter overall survival of JP02-infected mice as well as the unaltered survival by CD4-depletion is impaired pathogen recognition. Pathogen recognition by phagocytes is the first step in the immune response and is required for induction of CD4T cell-mediated immunity. Therefore, we tested whether mouse JAWSII DCs could recognize live H99 and JP02 cells in co-culture by measuring IL-6 released into the culture supernatant, a well-described response of immune cells in the presence of stimulants such as lipopolysaccharide. Whereas live H99 cells stimulated IL-6 production from JAWSII DCs, live JP02 cells did not (Figure 3A). However, it is possible that live JP02 cells suppress IL-6 production by cytotoxicity or by suppressive signals. To examine the first possibility, we cultured JAWSII DCs with heat-killed JP02 cells or H99 cells. Although heat-killed H99 cells still induced IL-6 production from JAWSII cells, heat-killed JP02 cells did not (Figure 3B). In addition, IL-6 production induced by H99 cells was not suppressed by co-inoculation with JP02 cells. Collectively, these results suggest that JP02 is not recognized by JAWSII DCs and that the hypervirulent *C. gattii* possesses molecular mechanism to evade recognition by host innate immune cells.

### Capsule and extracellular polysaccharides from H99 cells but not JP02 cells induce IL-6 production from murine dendritic cells

**Figure 4:**
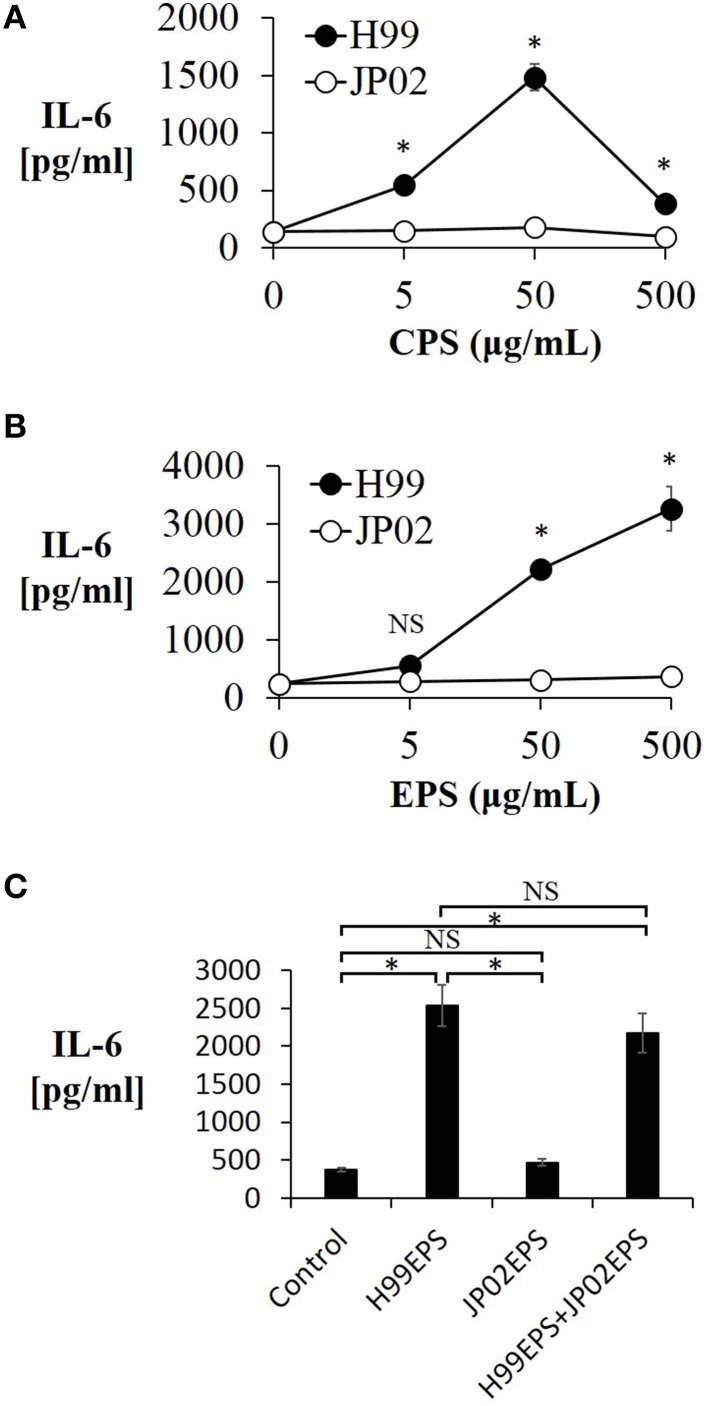
**Lower IL-6 release from JAWSII cells in response to CPS and EPS fractions of JP02 cells compared to CPS and EPS fractions of H99 cells**. JAWSII cells (2 × 10^5^ cells/mL) were incubated with CPS or EPS for 24 h, and supernatant obtained for ELISA. **(A)** CPS; **(B)** EPS, and **(C)** co-incubation with EPS from both H99 and JP02 (50 μg/mL, each). The experiments were performed in triplicate (means ± SD). Treatment group means were compared by the unpaired *t*-test, except for vs. CPS 500 μg/mL, which were compared by the Mann–Whitney U-test. ^*^P < 0.01; NS, not significant.

We next conducted experiments to identify molecular component(s) on the cell surface or secreted into the extracellular space that contribute to the evoked release of proinflammatory cytokines from DCs (and that may be less potent or absent in JP02 cells). Cryptococci express cell surface polysaccharides and previous studies have shown that cryptococcal polysaccharides induce production and release of proinflammatory cytokines (Delfino et al., 1996, 1997; Retini et al., 1996; Walenkamp et al., 1999). We thus extracted CPS and EPS from H99 and JP02 cells, and applied them to JAWSII DCs. The CPS extract from H99 cells induced IL-6 production from JAWSII DCs, although IL-6 production was attenuated at higher doses, i.e., 500 μg /mL (Figure 4A). On the other hand, CPS extract from JP02 cells did not induce measureable IL-6 production at any concentration tested. These results suggest that the JP02 CPS fraction is far less potent at inducing the inflammatory response than that from H99 cells. That higher H99 CPS concentrations also did not induce robust cytokine release may be explained by the formation of a filamentous matrix in the medium as revealed by microscopic observation (not shown).

Next, the cytokine-inducing capacity of JP02 EPS purified from culture supernatant was compared with that of H99 EPS. Amounts of EPS obtained per culture volume were similar between H99 and JP02 cells (Table 1). The EPS mixture purified from H99 induced IL-6 production in a dose-dependent manner, while that from JP02 did not (Figure 4B). To exclude the possibility that IL-6 in the supernatant resulted from lipopolysaccharide contamination in the EPS extract, the cationic antibiotic polymyxin B, which binds strongly to LPS, was added to the JAWSII culture with the EPS mixture. However, there was no effect of polymyxin B on EPS-induced IL-6 release (Figure S2). To determine whether the JP02 EPS mixture suppresses IL-6 production, JP02 EPS were mixed with H99 EPS and added to JAWSII DCs. However, the presence of JP02 EPS did not suppress IL-6 release stimulated by H99 EPS (Figure 4C). These results suggest that JP02 EPS are not recognized and so do not induce cytokine release from dendritic cells.

### Difference in glucuronoxylomannan O-acetylation between H99 and JP02 cells

**Figure 5:**
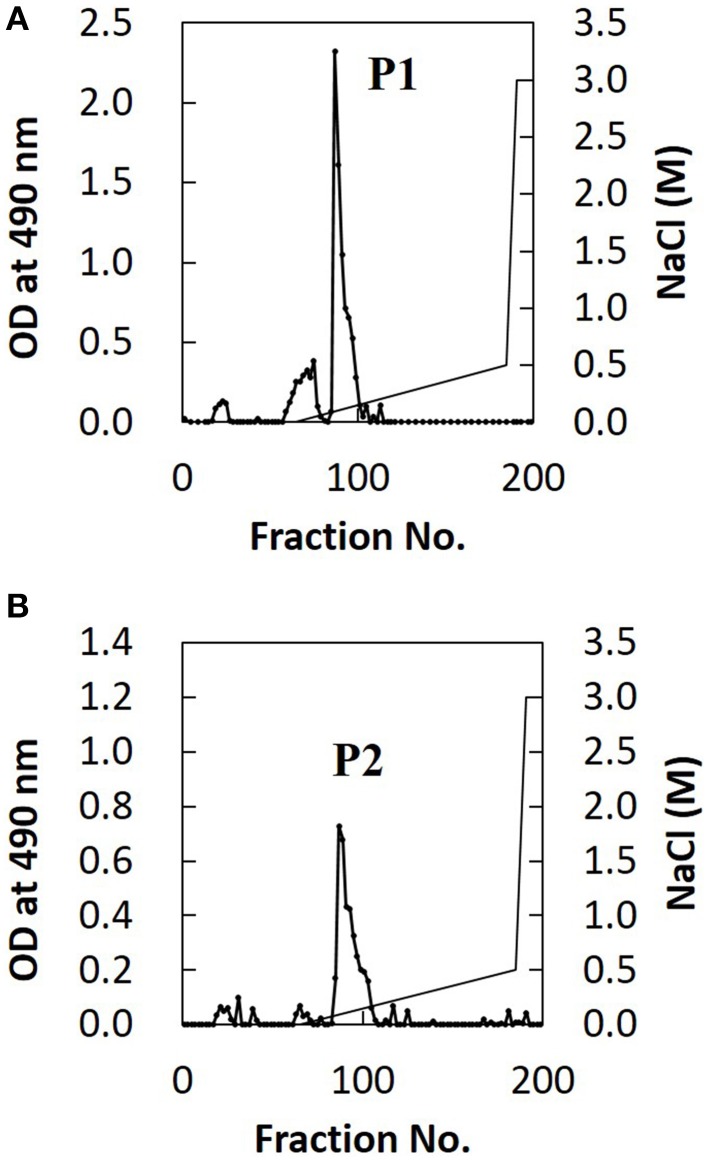
**Ion-exchange chromatography of EPS fractions from H99 and JP02 cells indicate similar compositions**. **(A)** H99 and **(B)** JP02. EPSs were dissolved in 10 mM Tris–HCl buffer, applied to a DEAE-Toyopearl column (180 mm × 25 mm ϕ), and eluted with a 600-mL linear gradient (0–0.5 M) of NaCl. Subfractions containing saccharides were monitored by the phenol–H_2_SO_4_ method.

The extracted EPS fraction is a mixture of several polysaccharides, so we performed anion column chromatography to separate individual components and identify those with structural differences between H99 and JP02. The EPS fractions from H99 and JP02 cells showed similar elution profiles and contained one major acidic subfraction (P). The P1 subfraction from H99 EPS and P2 subfraction from JP02 EPS were collected (Figures 5A,B). Monosaccharide composition analysis showed that both P1 and P2 contained glucuronic acid (GlcA), xylose (Xyl), and mannose (Man) (Figure S3; Table 1), indicating that they contain glucuronoxylomannan (GXM), a major cryptococcal capsule component (hereafter, P1 and P2 are designated as H99 GXM and JP02 GXM, respectively). There were only small differences in the monosaccharide composition between H99 GXM and JP02 GXM; specifically, the xylose content of JP02 GXM was higher than that of H99 GXM.

**Table 1:** **Compositions of major extracellular polysaccharides (EPS) fractions purified from H99 and JP02 strain (molar ratios)**.

**Fractions**	**Glucuronic acid^a^**	**Xylose^a^**	**Mannose^a^**	**O-Acetyl^b^**
H99 P1	1.0	2.4	4.1	1.5
JP02 P2	1.0	3.5	4.4	1.7

a *Calculated by high performance liquid chromatography (HPLC) analysis of aminobenzoic acid ethyl ester (ABEE)-labeled hydrolysate*.

c *Estimated by the ratio of acetyl-methyl resonances to anomeric protons of mannose backbone in the proton nuclear magnetic resonance (^1^H NMR) spectra*.

**Figure 6:**
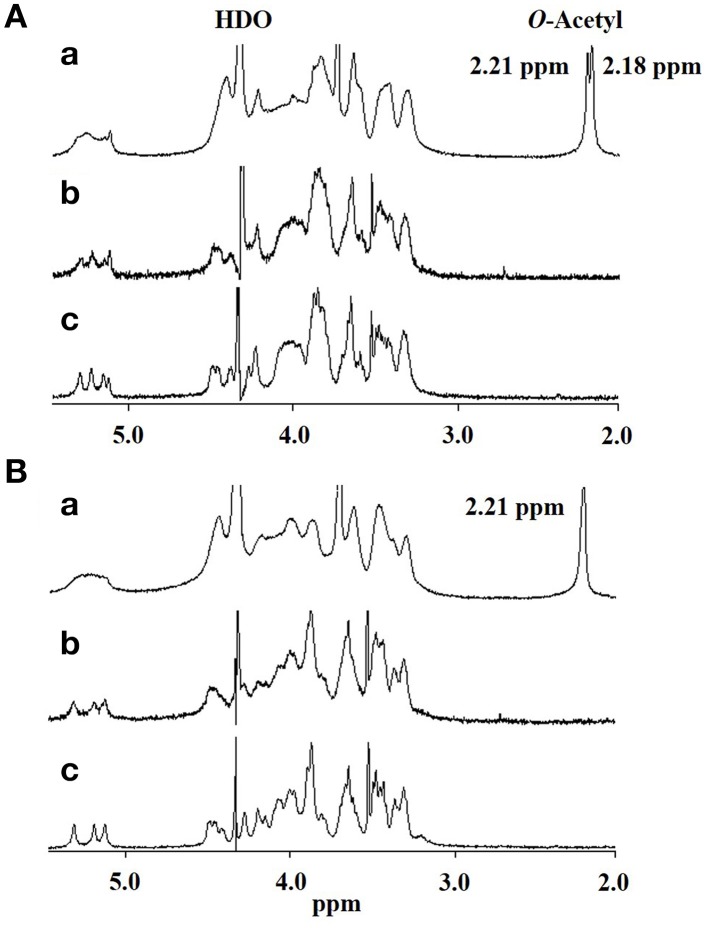
**500-MHz ^**1**^H NMR spectra of native and modified GXM purified from H99 and JP02 strains recorded in D_**2**_O at 70°C**. **(A)** H99 and **(B)** JP02. **(a)** native, **(b)** O-deacetylated, and **(c)** O-deacetylated and sonicated GXM.

Thus, if differences in GXM account for the difference in recognition by DCs, these must be at the level of molecular structure, so we performed further structural analysis by NMR. Proton nuclear magnetic resonance (^1^H NMR) analysis showed that the major difference between the spectrum of H99 GXM and JP02 GXM was the pattern of methyl protons indicating *O*-acetyl groups (Figure 6). The spectrum of H99 GXM contains 2 methyl protons of *O*-acetyl groups, whereas that of JP02 GXM contains only one methyl proton (Figures 6Aa,Ba). The methyl proton detected at 2.21 ppm in JP02 GXM was also detected in H99 GXM, but H99 GXM had one additional *O*-acetyl group detected at 2.18 ppm. However, acetyl contents were almost the same between H99 GXM and JP02 GXM as estimated by the ratio of acetyl-methyl resonances to anomeric protons of the mannose backbone in the ^1^H NMR spectra (Table 1).

**Figure 7:**
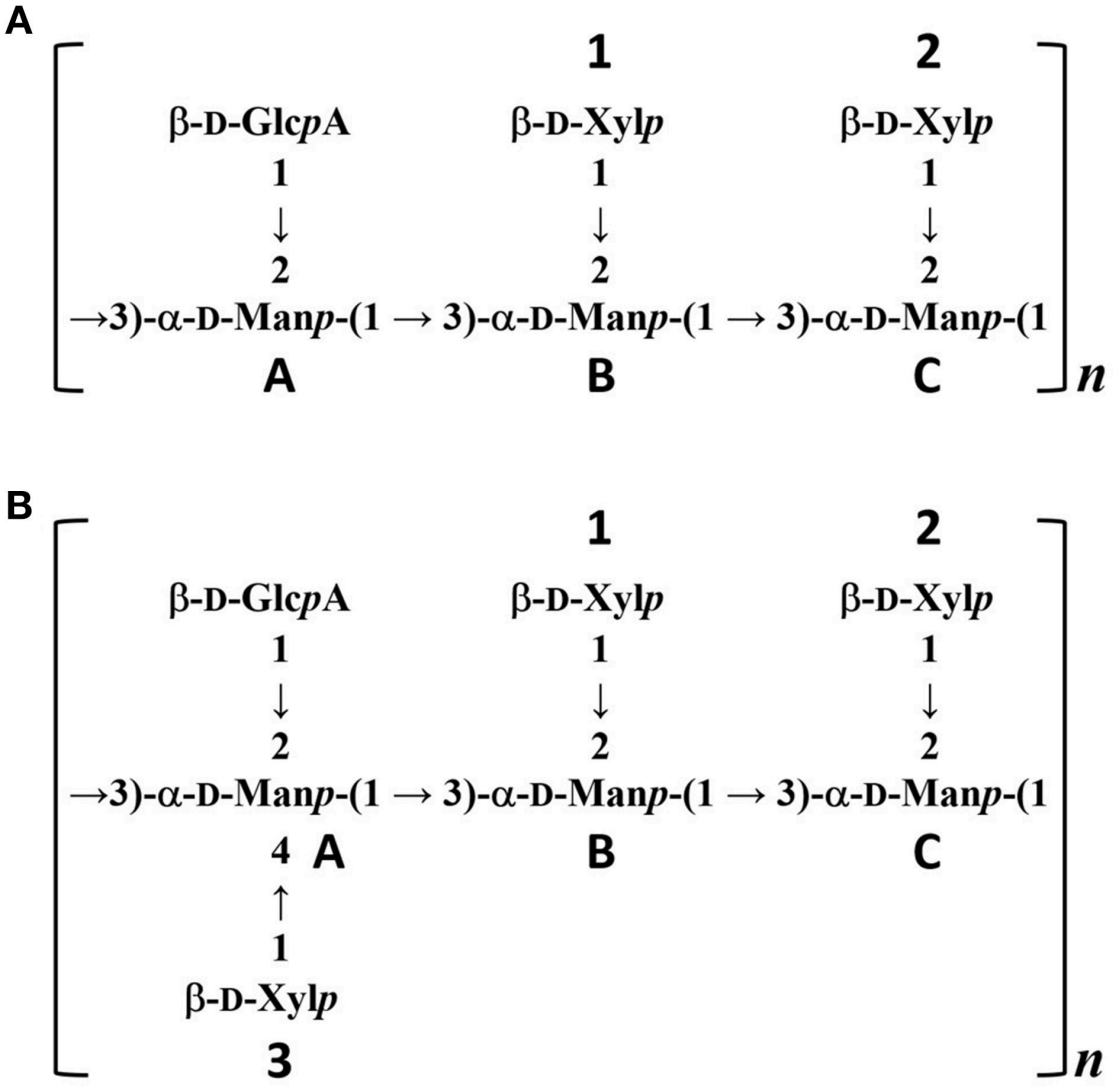
**Predicted structures of O-deacetylated GXMs purified from H99 and JP02 strains**. **(A)** H99 and **(B)** JP02.

To confirm the glycan structure of H99 and JP02 GXMs, they were deacetylated by weak alkali hydrolysis and sonicated to reduce the molecular weight. The ^1^H NMR spectra of deacetylated and sonicated H99 GXM and JP02 GXM (Figures 6Ac,Bc) were almost the same as those previously reported from serotype A and B (Serotype B is now called *C. gattii*), respectively (Turner and Cherniak, 1991; Sheng and Cherniak, 1997). ^1^H chemical shifts of anomeric protons were close to values reported previously (Table 2). These results suggest that H99 GXM has the glycan structure of serotype A, consistent with chemotype Chem4 (structure reporter group (SRG) M2: 100%), while JP02 GXM has that of serotype B, consistent with chemotype Chem7 (SRG M3: 100%) (Cherniak et al., 1998). The predicted glycan structures of GXMs purified from H99 and JP02 indicate that the JP02 GXM has one additional xylose residue (Xyl^3^ residue) on the mannose substituted by glucuronic acid (Man^A^ residue) in the repeating unit compared to the H99 GXM (Figure 7). Mannan backbone of the JP02 GXM seems to be more shielded from the outer environments compared with that of the H99 GXM.

**Table 2:** **^**1**^H chemical shifts (δ) of anomeric protons in the deacetylated and sonicated glucuronoxylomannan (GXM) isolated from H99 and JP02 (recorded in D_**2**_O at 70°C)**.

**GXM**	**Man^A^**	**Man^B^**	**Man^C^**	**GlcA**	**Xyl^1^**	**Xyl^2^**	**Xyl^3^**
H99	5.25	5.32	5.18	4.47	4.50	4.40	–
JP02	5.21	5.33	5.14	4.47	4.50	4.29	4.42

*The nomenclature of sugar residues is displayed in the predicted structures of GXMs shown in Figure 7. H99 GXM had one more anomeric proton detected at 5.14 ppm (Figure 6Ac). It was also detected in the GXM of the other serotype A strain, but was not assigned previously (Sheng and Cherniak, 1997)*.

### O-acetylation pattern of GXM may be involved in induction of inflammatory cytokine production from dendritic cells

**Figure 8:**
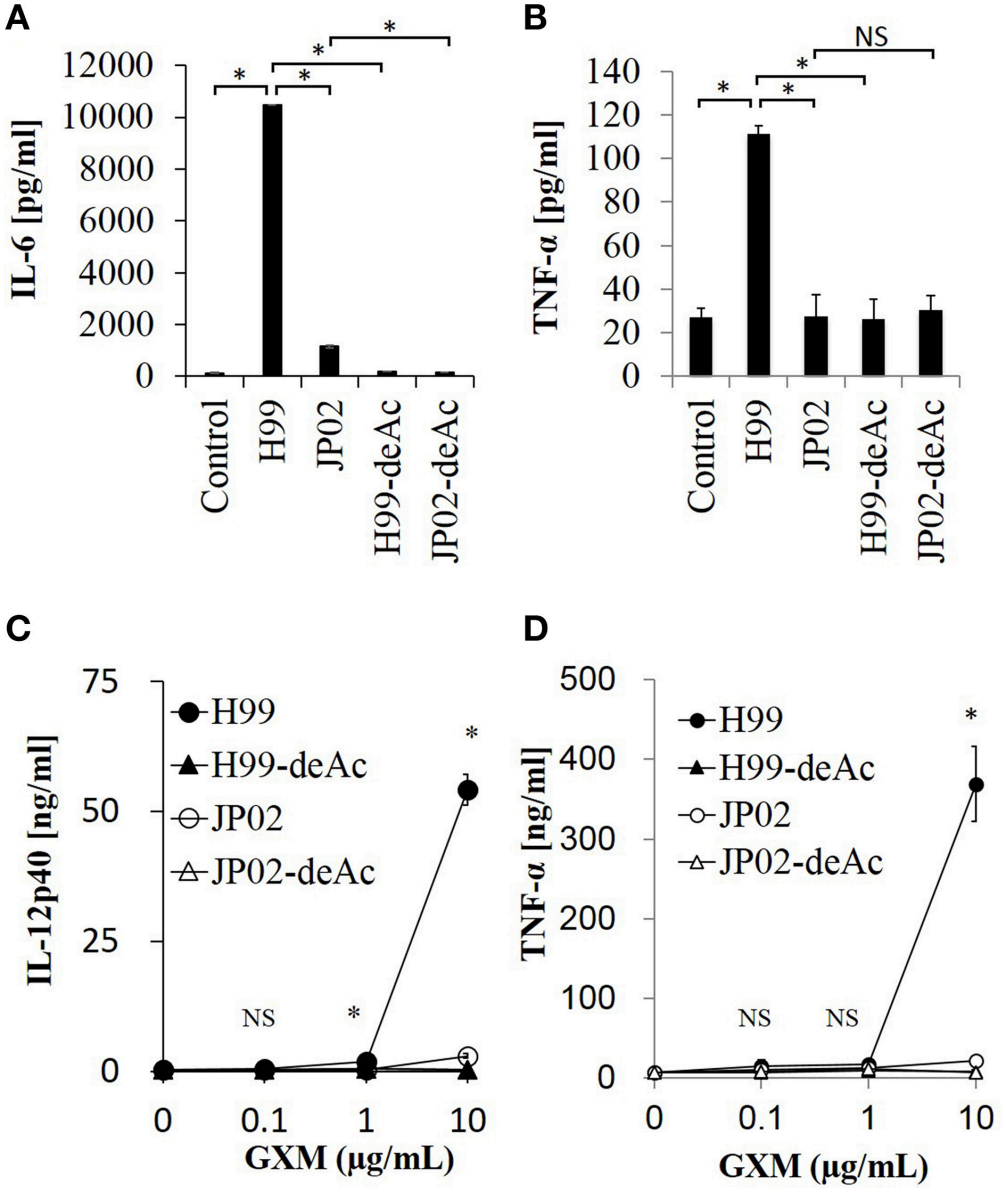
**Cytokine release from dendritic cells in response to native GXM purified from H99 and JP02 strains and the corresponding O-deacetylated products**. IL-6 **(A)** and TNF-α **(B)** release from JAWSII cells (2 × 10^5^cells/mL) stimulated by GXMs (50 μg/mL). IL-12p40 **(C)** and TNF-α **(D)** release from BMDCs (1 × 10^6^cells/mL) stimulated by GXMs. The experiments were performed in triplicate (means ± SD). Data sets in **(A)** or **(B)** were compared by the unpaired *t*-test, except for vs. control in **(A)**, which were compared by the Mann–Whitney U-test. Treatment group means of **(C)** or **(D)** were compared to H99 GXM by the unpaired *t*-test, except for vs. JP02 at 10 μg/mL in **(C)**, which were compared by Welch's *t*-test. ^*^P < 0.01; NS, not significant. Representative data from 3 independent experiments are shown.

To test whether the O-acetylation pattern in GXM mediates the recognition by DCs and thus accounts for the differential recognition of H99 and JP02, we compared the induction efficacy of native (intact) to deacetylated GXM. The H99 and JP02 GXMs were deacetylated with weak alkali treatment and confirmed by ^1^H NMR analysis (Figures 6Ab,Bb). Intact H99 GXM induced robust IL-6 production from JAWSII DCs, while intact JP02 GXM induced only a small amount of IL-6 release (Figure 8A). In contrast, IL-6 production was almost completely eliminated by deacetylation of GXM in both strains. Intact H99 GXM also induced TNF-α production from JAWSII DCs, while intact JP02 GXM and deacetylated GXM of both strains did not induce significant production of this cytokine (Figure 8B).

Finally, we evaluated the cytokine-inducing capacity of these GXMs on primary DCs. We used BMDCs of C57BL/6J mice and focused on production of interleukin-12 (IL-12), an important cytokine for the protection of mice against *C. neoformans* infection (Kawakami et al., 1996). Intact H99 GXM, but not intact JP02 GXM, induced IL-12p40 release from BMDCs in a dose-dependent manner (Figure 8C). Furthermore, IL-12p40 release from BMDCs was almost completely absent in response to deacetylated H99 GXM, consistent with results from JAWSII DCs. Similarly, intact H99 GXM induced TNF-α release from BMDCs, while intact JP02 GXM and deacetylated GXM of both strains did not induce release of this cytokine (Figure 8D). These results suggest that the O-acetylation pattern in GXM may play a key role in the recognition of *Cryptococcus* by dendritic cells and subsequent induction of cytokine release.

## Discussion

The histopathological features of *C. gattii* JP02 infection characterized in this study are similar to infection by R265, the highly virulent *C. gattii* strain clinically isolated during the Canadian outbreak (Cheng et al., 2009; Leongson et al., 2013; Okubo et al., 2013). We found a shorter survival time following *C. gattii* JP02 infection compared with that following *C. neoformans* H99 infection. Moreover, CD4 depletion shortened the survival of mice infected with *C. neoformans* H99 but had no effect on mortality of CD4-depleted mice infected with *C. gattii* JP02. These results suggest that JP02 is highly virulent even in mice with functional CD4-positive cells. This difference of virulence between *C. gattii* and *C. neoformans* is consistent with the fact that *C. gattii* infection frequently occurs in relatively healthy people while cryptococcosis due to *C. neoformans* tends to occur more frequently in immunocompromised hosts, especially HIV-infected patients (Galanis et al., 2009, 2010). Leongson et al. showed that the similar phenomena using CD4C/HIV^MutA^ transgenic mice (which exhibit a preferential depletion of CD4+ T cells) infected with H99 or R265 (Leongson et al., 2013). It was demonstrated that HIV-1 transgene expression augmented susceptibility to *C. neoformans* but not *C. gattii* infection, and reduced the pulmonary inflammatory cell response by both depletion of immune cells and diminished production of chemokines. However, the factors responsible for the differences in immune response between *C. neoformans* and *C. gattii* were still unclear. Then, we speculated that the reason for the higher virulence of JP02 may be lack of recognition by host immune cells and concomitant evasion of phagocytosis. We demonstrate that the higher virulence of JP02 compared to H99 stems from a structural difference in the capsular GXM, which greatly reduces the capacity of JP02 GXM to induce cytokine release from local lung DCs. Thus, this study identifies the GXM as a key factor controlling recognition of *Cryptococcus* by cells contributing to innate immunity and thus the pathogenicity of different *Cryptococcus* species/strains.

We first showed that H99 induced granuloma formation in the murine lung, while JP02 did not. The less robust protective inflammatory response in lungs of JP02-infected mice may account for the higher virulence of *C. gattii* even in immunocompetent hosts. We speculated that there is an impairment of pathogen recognition for JP02. To identify the molecules (s) conferring recognition (possibly aberrant in JP02), we compared IL-6 release from cultured mouse JAWSII DCs induced by H99 and JP02 cells and found that only H99 cells induced this response, suggesting that hypervirulent *C. gattii* possesses molecular mechanism to evade recognition. Further experiments with intact cells and various cellular fractions identified GMX as the major determinant of host recognition.

Virulence of cryptococcal pathogens is thought to be partly dependent on the capsule. It has been shown that the capsule of *C. neoformans* suppresses the host innate immunity by comparing encapsulated strain to acapsular mutant. Highly encapsulated strains of cryptococci resists for phagocytosis by immunocyte compared to the acapsular mutants (Buchanan and Murphy, 1998). An acapsular mutant of *C. neoformans* activated and facilitated maturation of human DC, whereas an encapsulated strain interfered with DC activation and maturation (Vecchiarelli et al., 2003). Siddiqui et al. demonstrated that IL-6 plays an important role in resistance to *C. neoformans* infection, and encapsulated strain of *C. neoformans* induce lower IL-6 production from human peripheral blood mononuclear cells than acapsular mutant (Siddiqui et al., 2006).

The capsule is composed primarily of two polysaccharides, GXM (90–95%) and galactoxylomannan (GalXM, 5–8%), in addition to a smaller proportion of mannoproteins (MP, <1%) (Zaragoza et al., 2009). GXM is the major component of the capsule, and it is also secreted abundantly into the ambient environment (Cherniak and Sundstrom, 1994). The GXM of *C. neoformans* has an important role in abolishing host immunity by inhibiting binding of antibodies and complement C3 (Young and Kozel, 1993; Cherniak and Sundstrom, 1994). Yauch et al. has shown that GXM extracted from *C. neoformans* and *C. gattii* inhibits T-cell proliferation directly (Yauch et al., 2006). On the other hands, the GXM of *C. neoformans* can induce expression and release of inflammatory cytokines, such as TNF-α and IL-6, from human peripheral blood mononuclear cells (Walenkamp et al., 1999), neutrophils (Retini et al., 1996), leukocytes (Delfino et al., 1996), and monocytes (Delfino et al., 1997). As H99 and JP02 strains were encapsulated in the lungs of infected mice, we focused on differences in cell surface and secreted polysaccharides. We demonstrated that the CPS, EPS, and GXM fractions from JP02 induced lower levels of proinflammatory cytokine secretion by DCs than the same fractions of H99 cells. These differences were not due to suppression of cytokine release, as H99 capsule-induced release was the same in the presence and absence of JP02 capsule components, but rather due to lower recognition of JP02 capsule components by DCs (Figures 3, 4). JP02 GXM also did not induce the production of IL-12, an important cytokine for protection of mice against *C. neoformans* infection, by initiating Th1 immune responses (Kawakami et al., 1996). Thus, lower induction of DC IL-12p40 production by JP02 GXM may contribute to reduced activation of cellular immunity and account for the higher virulence of JP02 in immunocompetent hosts. Consistent with this result, CD4 depletion did not influence the survival or fungal burden in mice infected with JP02 (Figure 1).

To clarify the relationship between GXM structure and inflammatory cytokine-inducing activity, we prepared deacetylated GXM and showed that the O-acetylation pattern affects cytokine release. An aberrant O-acetyl residue in JP02 GXM detected at 2.18 ppm in H99 GXM by ^1^H NMR may contribute to a conformational change allowing JP02 cells to evade recognition by innate immune cells and thus escape phagocytic elimination by the host. Indeed, O-acetyltransferase-deficient mutants of *C. neoformans* were reported to be hypervirulent against mice compared to wild type *C. neoformans*, supporting our hypothesis (Janbon et al., 2001). The O-acetylation of *C. neoformans* GXM is involved in the binding of antibody (Kozel et al., 2003) and the interference of neutrophil migration by GXM (Ellerbroek et al., 2004).

The locations of the O-acetyl groups in *C. neoformans* GXM had not been determined previously, except for one strain of *C. neoformans* serotype D (Janbon et al., 2001). According to that report, the methyl proton detected at 2.18 ppm in the ^1^H NMR spectrum is the O-acetyl group bound to the C6 position of the mannose residue substituted by a glucuronic acid at the C2 position. The JP02 GXM has one additional xylose residue substituted at the C4 position of that mannose residue compared with H99 GXM (Man^A^ residue displayed in Figures 7A,B). One possible reason for the defect of this acetyl residue in the JP02 GXM may be the spatial regulation around the mannose residue to access the O-acetyltransferase. However, it remains unclear why the O-acetyl residue detected in JP02 GXM at 2.20 ppm by ^1^H NMR is not recognized by DCs. We speculate that DCs do not recognize the O-acetylated mannose residue directly, but distinguish the conformational change in the GXM molecule arising from the difference in O-acetylation pattern. While the pathogenicity of *C. gattii* cannot be explained entirely by this aberrant acetyl group, our results strongly suggest that it does contribute to the hypervirulence of JP02.

Two other capsular components GalXM and MP also play important roles in immune response. It was shown that MP is highly immunogenic (Levitz and Specht, 2006) and that GalXM induces more TNF-α release from whole blood cultures compared to GXM (Delfino et al., 1996). Compositional and NMR analysis of the GXM fraction purified in this study indicate that the GXM was separated successfully from the other capsule components, such as GalXM and MP. Future studies on assessment of the immunoreactivity of *C. gattii* GalXM and MP may also contribute to unveil the high virulence of *C. gattii*.

In conclusion, this is the first report suggesting a possible role for the GXM O-acetylation pattern in cryptococcal recognition by dendritic cells, and our data indicate that the highly virulent *C. gattii* JP02 strain evades the innate immune response by altering the structure of GXM. In this study, we focused on two cryptococcal strains to perform extensive structural and immunological analysis. Although further studies are needed to determine whether the O-acetylation pattern of GXM is important for virulence of other cryptococcal strains, this work identifies one possible molecular mechanism determining *Cryptococcus* virulence in healthy hosts. Investigation of host factors, such as pattern recognition receptors that can distinguish the difference in the GXM O-acetylation pattern among strains, is currently in progress.

## Author contributions

MU, YKa, HF, TS, HO, YKi, and YM conceived and designed the experiments. MU, YKa, KU, and TA performed the experiments. YO and KS performed the histological analyses. MU wrote the paper.

## Funding

This work was partly supported by a grant from the Ministry of Health, Labour and Welfare of Japan (H20-shinkou-ippan-012, H22-shinkou-ippan-008, H23-shinkou-ippan-018, H25-shinkou-shitei-002, and H26-shinkougyousei-shitei-002), by the Research Program on Emerging and Re-emerging Infectious Diseases from the Japan Agency for Medical Research and Development, AMED, and by a Grant-in-aid for Scientific Research (21390305, 24791032, and 15K21644) from the Ministry of Education, Culture, Sports, Science, and Technology of Japan, and by the NOVARTIS Foundation (Japan) for the Promotion of Science.

### Conflict of interest statement

The authors declare that the research was conducted in the absence of any commercial or financial relationships that could be construed as a potential conflict of interest.
