# Supplementary material for: Evasion of Innate Immune Responses by the Highly Virulent Cryptococcus gattii by Altering Capsule Glucuronoxylomannan Structure
Source: Front Cell Infect Microbiol. 2016 Jan 6;5:101. doi: 10.3389/fcimb.2015.00101 (PMC4701946; doi:10.3389/fcimb.2015.00101)
Supplement: Supplementary file 1 [file Presentation1.PPTX]

## Slide 1
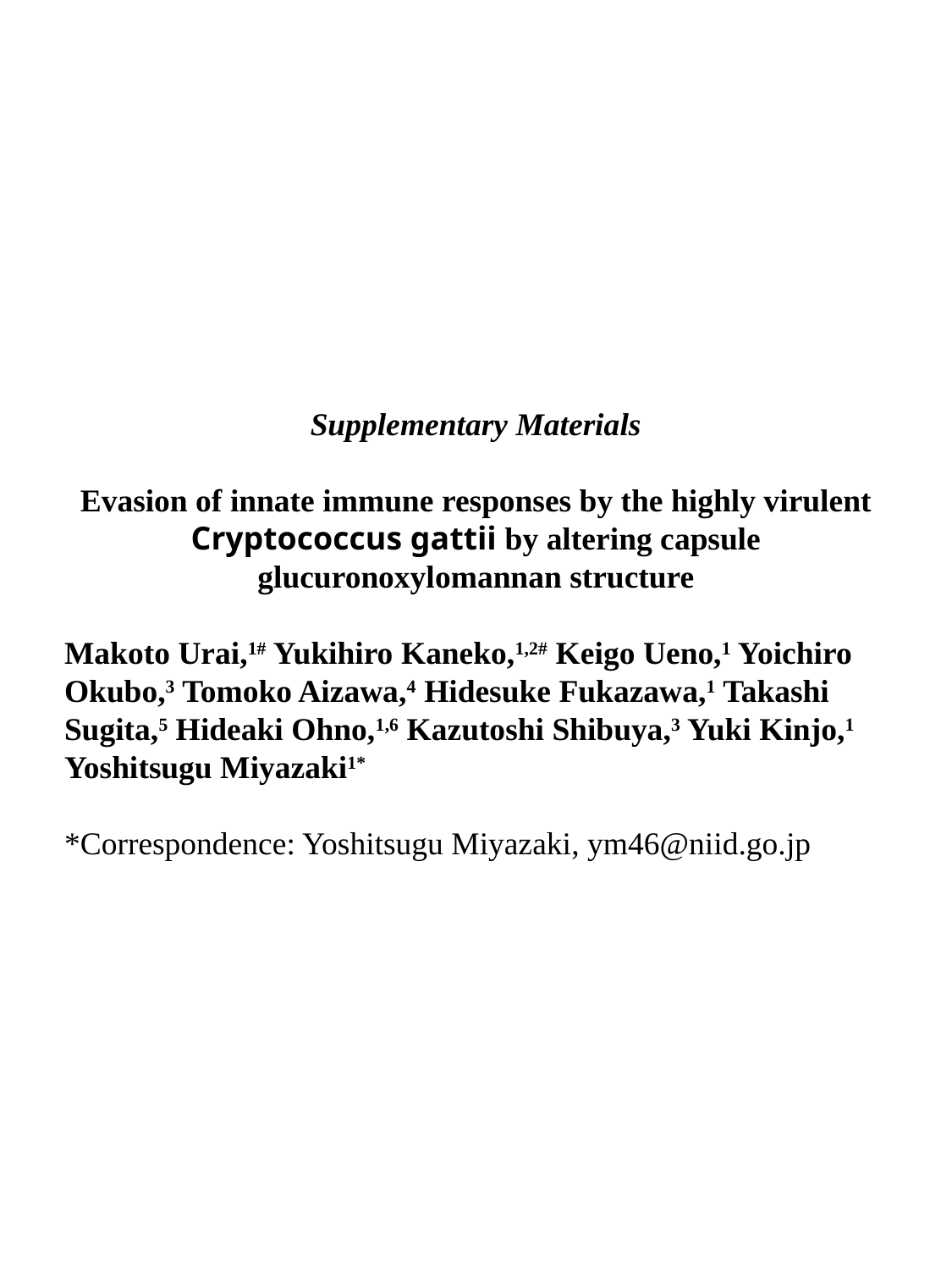

Supplementary Materials
Evasion of innate immune responses by the highly virulent Cryptococcus gattii by altering capsule glucuronoxylomannan structure
Makoto Urai,1# Yukihiro Kaneko,1,2# Keigo Ueno,1 Yoichiro Okubo,3 Tomoko Aizawa,4 Hidesuke Fukazawa,1 Takashi Sugita,5 Hideaki Ohno,1,6 Kazutoshi Shibuya,3 Yuki Kinjo,1 Yoshitsugu Miyazaki1*
*Correspondence: Yoshitsugu Miyazaki, ym46@niid.go.jp

## Slide 2
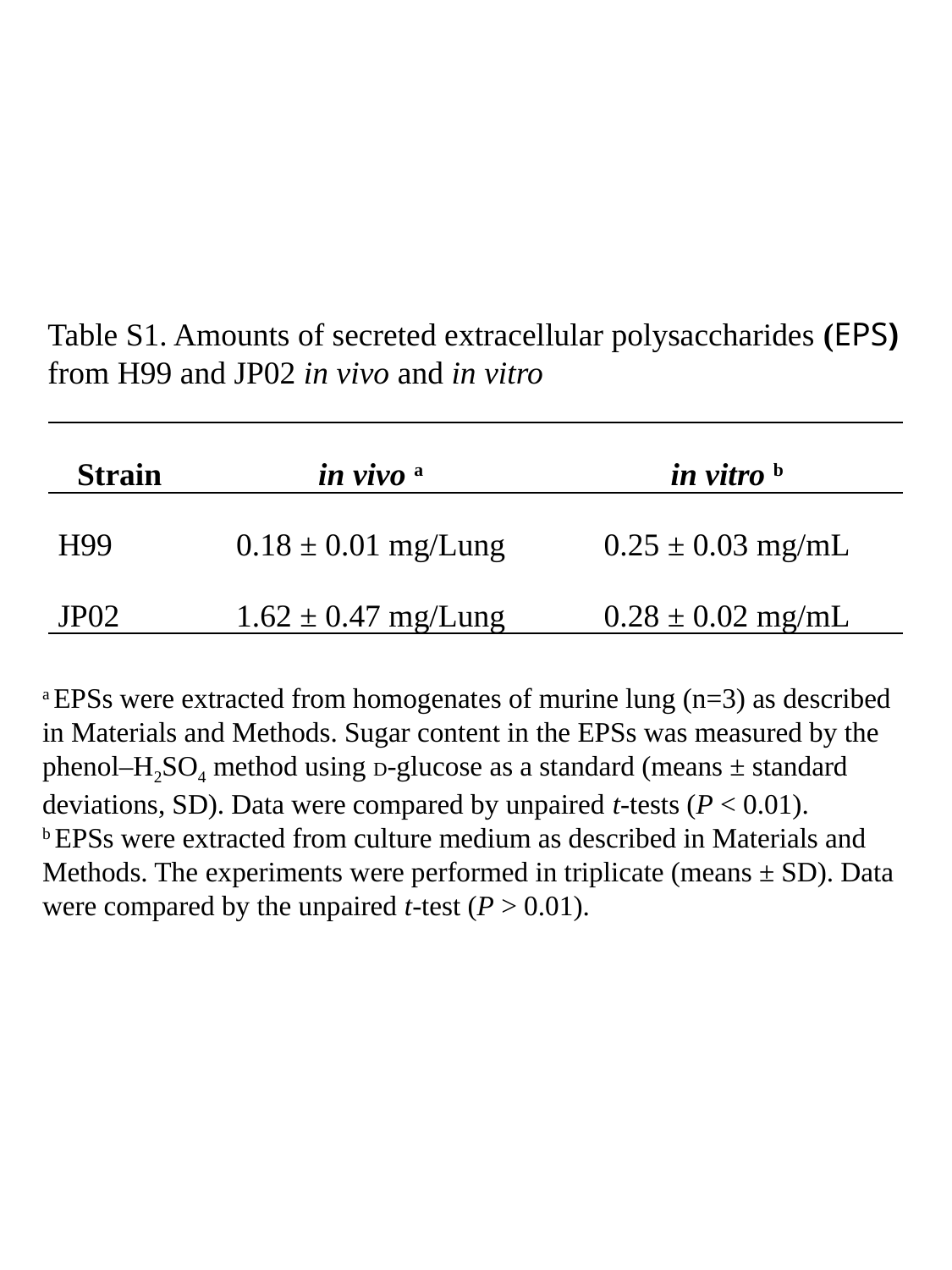

Table S1. Amounts of secreted extracellular polysaccharides (EPS) from H99 and JP02 in vivo and in vitro
| Strain | in vivo a | in vitro b |
| --- | --- | --- |
| H99 | 0.18 ± 0.01 mg/Lung | 0.25 ± 0.03 mg/mL |
| JP02 | 1.62 ± 0.47 mg/Lung | 0.28 ± 0.02 mg/mL |
a EPSs were extracted from homogenates of murine lung (n=3) as described in Materials and Methods. Sugar content in the EPSs was measured by the phenol–H2SO4 method using D-glucose as a standard (means ± standard deviations, SD). Data were compared by unpaired t-tests (P < 0.01).
b EPSs were extracted from culture medium as described in Materials and Methods. The experiments were performed in triplicate (means ± SD). Data were compared by the unpaired t-test (P > 0.01).

## Slide 3
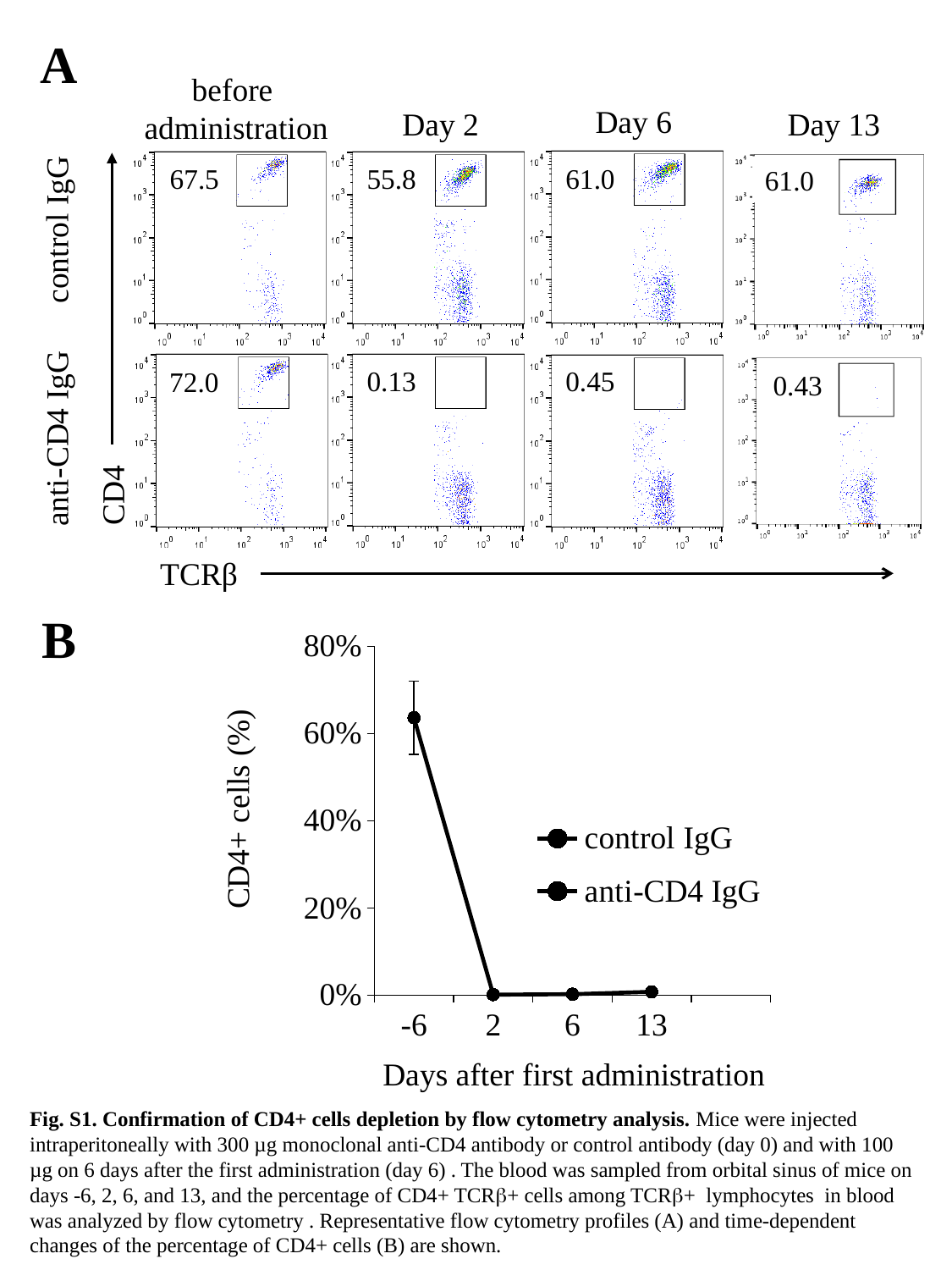

A
before
administration
Day 6
Day 2
Day 13
67.5
55.8
61.0
61.0
control IgG
0.13
0.45
72.0
0.43
anti-CD4 IgG
CD4
TCRβ
B
### Chart
| Category | control IgG | anti-CD4 IgG |
|---|---|---|
| -6 | 0.672151288218003 | 0.636299112801014 |
| 2 | 0.54395084580486 | 0.00121117459873005 |
| 6 | 0.610558562801977 | 0.00223713646532439 |
| 13 | 0.532702026892342 | 0.00772195220154727 |CD4+ cells (%)
Days after first administration
Fig. S1. Confirmation of CD4+ cells depletion by flow cytometry analysis. Mice were injected intraperitoneally with 300 µg monoclonal anti-CD4 antibody or control antibody (day 0) and with 100 µg on 6 days after the first administration (day 6) . The blood was sampled from orbital sinus of mice on days -6, 2, 6, and 13, and the percentage of CD4+ TCRb+ cells among TCRb+ lymphocytes in blood was analyzed by flow cytometry . Representative flow cytometry profiles (A) and time-dependent changes of the percentage of CD4+ cells (B) are shown.

## Slide 4
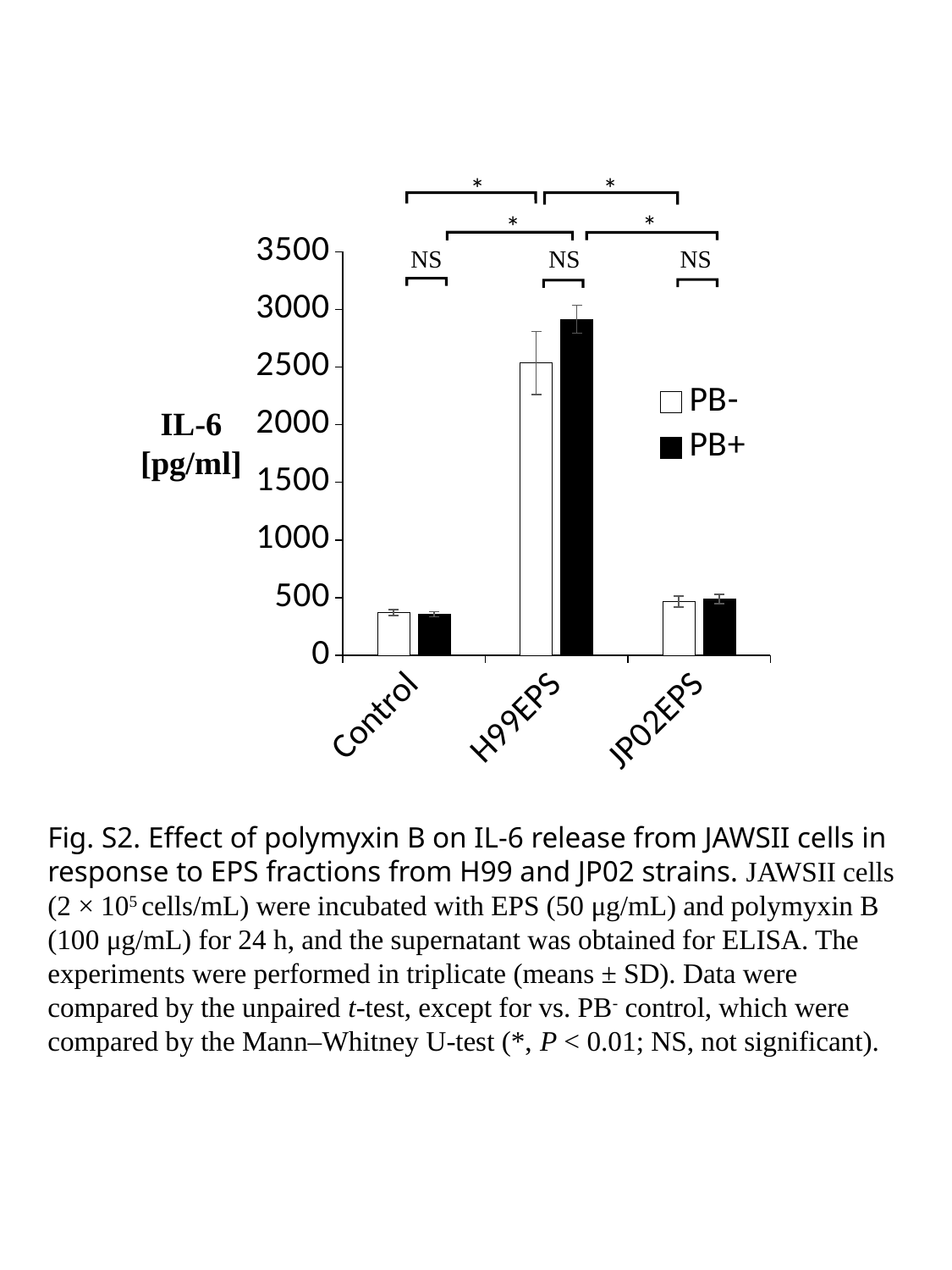

*
*
*
*
### Chart
| Category | PB- | PB+ |
|---|---|---|
| Control | 370.17610876226667 | 356.7090405944666 |
| H99EPS | 2533.3199594998 | 2913.8107691644 |
| JP02EPS | 466.15091430193337 | 488.8318166898667 |NS
NS
NS
IL-6
[pg/ml]
Fig. S2. Effect of polymyxin B on IL-6 release from JAWSII cells in response to EPS fractions from H99 and JP02 strains. JAWSII cells (2 × 10^5^ cells/mL) were incubated with EPS (50 μg/mL) and polymyxin B (100 μg/mL) for 24 h, and the supernatant was obtained for ELISA. The experiments were performed in triplicate (means ± SD). Data were compared by the unpaired t-test, except for vs. PB- control, which were compared by the Mann–Whitney U-test (*, P < 0.01; NS, not significant).

## Slide 5
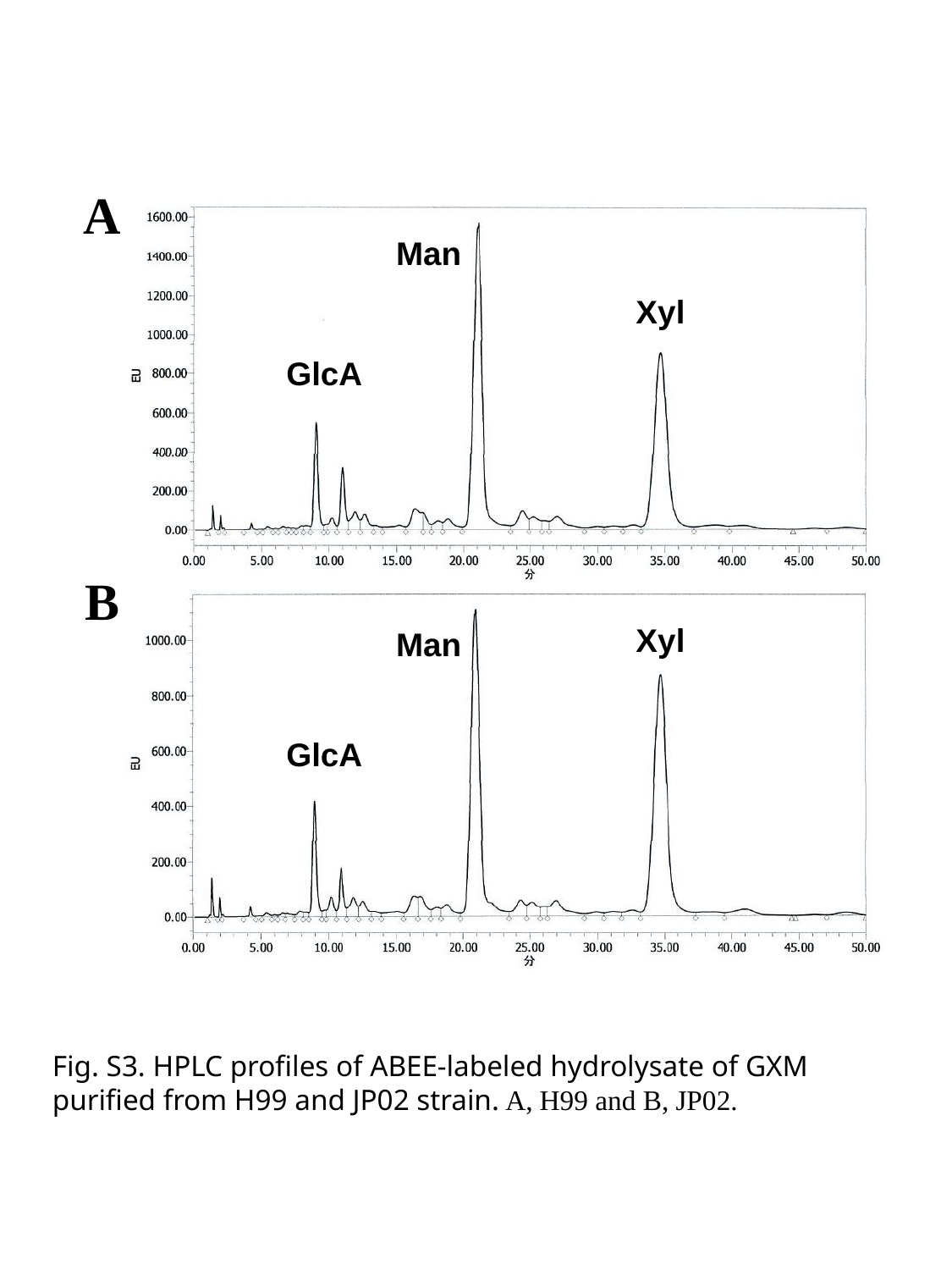

A
Man
Xyl
GlcA
B
Xyl
Man
GlcA
Fig. S3. HPLC profiles of ABEE-labeled hydrolysate of GXM purified from H99 and JP02 strain. A, H99 and B, JP02.
